# Analysis of the genotype–phenotype correlation in isovaleric acidaemia: A case report of long-term follow-up of a chinese patient and literature review

**DOI:** 10.3389/fneur.2022.928334

**Published:** 2022-07-28

**Authors:** Xingmiao Liu, Xinquan Liu, Wenxuan Fan, Zhongbin Zhang, Peiyuan Zhang, Xiaojun Liu, Meifang Lei, Qing Li, Xiaoli Yu, Dong Li

**Affiliations:** ^1^Department of Pediatric Neurology, Tianjin Children's Hospital/Tianjin University Children's Hospital, Tianjin, China; ^2^School of Precision Instrument and Optoelectronics Engineering, Tianjin University, Tianjin, China

**Keywords:** isovaleric acidaemia, isovaleryl-CoA dehydrogenase (IVD), inherited metabolic disease, genotype-phenotype correlation, case report

## Abstract

**Background:**

Isovaleric acidaemia (IVA), characterized by an acute metabolic crisis and psychomotor delay, is a rare inherited metabolic disease caused by a deficiency in isovaleryl-CoA dehydrogenase (IVD).

**Methods:**

We report the case of a Chinese patient with IVA who was admitted to Tianjin Children's Hospital and followed up for 8 years. Genetic analysis of the patient and his parents was conducted using the whole-exome sequencing and Sanger sequencing. We searched for similar reported cases in the PubMed and Wanfang databases using the term “isovaleric acidaemia,” reviewed the related literature to obtain a summary of the clinical and genetic characteristics, and analyzed the genotype–phenotype correlations.

**Results:**

The patient presented with encephalopathic symptoms, such as vomiting, lethargy, and somnolence. We identified compound heterozygous variants of the *IVD* gene, including the unreported variant c.224A>G (p.Asn75Ser) and the reported variant c.1195G>C (p.Asp399His). The child was prescribed a low-protein diet supplemented with L-carnitine. During the 8-year follow-up, no metabolic disorder or encephalopathic symptoms recurred. At present, the child is 11 years of age and has normal mental and motor performance. Another 154 cases identified in 25 relevant references were combined with this case, resulting in a sample of 155 patients, including 52 asymptomatic patients, 64 with neonatal onset, and 39 with the chronic intermittent disease with onset from ages of 1 month to 10 years (median age, 2 years). Among articles that reported sex, the male-to-female ratio was 1:1.06. The cardinal symptoms included vomiting, lethargy, “sweaty foot” odor, poor feeding, developmental delay, and epilepsy. The proportion of variants in regions 123–159 and 356–403 of the IVD protein was greater in symptomatic patients than in asymptomatic patients. Conversely, in asymptomatic patients, the proportion of variants in the 282–318 region was greater than in symptomatic patients.

**Conclusion:**

This case report describes an unreported variant c.224A>G (p.Asn75Ser) of the *IVD* gene, and summarizes previously reported cases. Furthermore, the correlation between the genotype and clinical phenotype of IVA is analyzed to improve the understanding of this disease.

## Introduction

Isovaleric acidaemia (IVA) is a rare inherited metabolic disease caused by a deficiency in isovaleryl-CoA dehydrogenase (IVD) (1), which catalyses the oxidation of isovaleryl-CoA to 3-methylcrotonyl-CoA during the third step of leucine catabolism (2). The clinical manifestations of IVA include paroxysmal vomiting, lethargy or altered mental status, epilepsy, poor feeding, developmental delay, severe metabolic acidosis, hyperammonaemia, ketosis, hyper- or hypoglycaemia, and cytopenia. A typical “sweaty foot” odor is often found in the acute phase. The absence or delay of treatment can result in death or developmental delay. IVA can be categorized into three subtypes: (1) Acute neonatal type: disease presents in the first 2 weeks after birth, including severe metabolic acidosis and encephalopathy, often accompanied by leukopenia, neutropenia, thrombocytopenia, electrolyte disturbances, and hypo- or hyperglycaemia. The patient may experience coma or death if not properly treated (3). (2) Chronic intermittent type: findings include psychomotor delay and paroxysmal metabolic disorder. During the metabolic crisis, clinical manifestations are similar to those of the acute neonatal type (4). (3) Asymptomatic type: findings include mild biochemical abnormalities (5).

Isovaleryl-CoA dehydrogenase is a mitochondrial flavinase and member of the acyl-CoA dehydrogenase family. Its precursor is encoded within the nuclear genome, and the precursor peptides are synthesized in the cytoplasm and then transported into the mitochondria, where the leading N-terminal sequence is removed by proteolysis to form a mature homologous tetramer. Each monomer contains a noncovalently but tightly bound flavin adenine dinucleotide (FAD) molecule (6). The lengths of its precursor protein and mature sequence are 424 and 394 amino acid residues, respectively (7). The human *IVD* gene is located on chromosome 15q14-q15 and is 15 kb in length, consisting of 12 exons and 11 introns (8). In previous reports, more than 130 variants have been described; however, the relationship between genotype and phenotype remains unclear. Few studies have been conducted in China, and most cases have been reported without systematic analysis of disease characteristics. This paper reports the case of a child with IVA admitted to Tianjin Children's Hospital and followed up for 8 years. The clinical phenotype, genetic characteristics, and laboratory examination findings of the child are summarized, and the correlation between the genotype and clinical phenotype of IVA is analyzed through a literature review to deepen our understanding of this genetic disease.

## Clinical report

### Clinical manifestations

The patient, an 11-year-old boy with non-consanguineous Chinese parents, was born at term through an uneventful delivery and with normal birth parameters. The boy was born from a first pregnancy, and his birth weight was 3.35 kg. Developmental milestones were normal. When he was 3 years of age, after 2 days of fever, he presented with severe and frequent vomiting, up to 15 times per day, accompanied by lethargy and somnolence. The family history included no specific genetic diseases. A physical examination revealed no abnormalities. Psychomotor development was normal.

### Auxiliary examination

**Figure 1:**
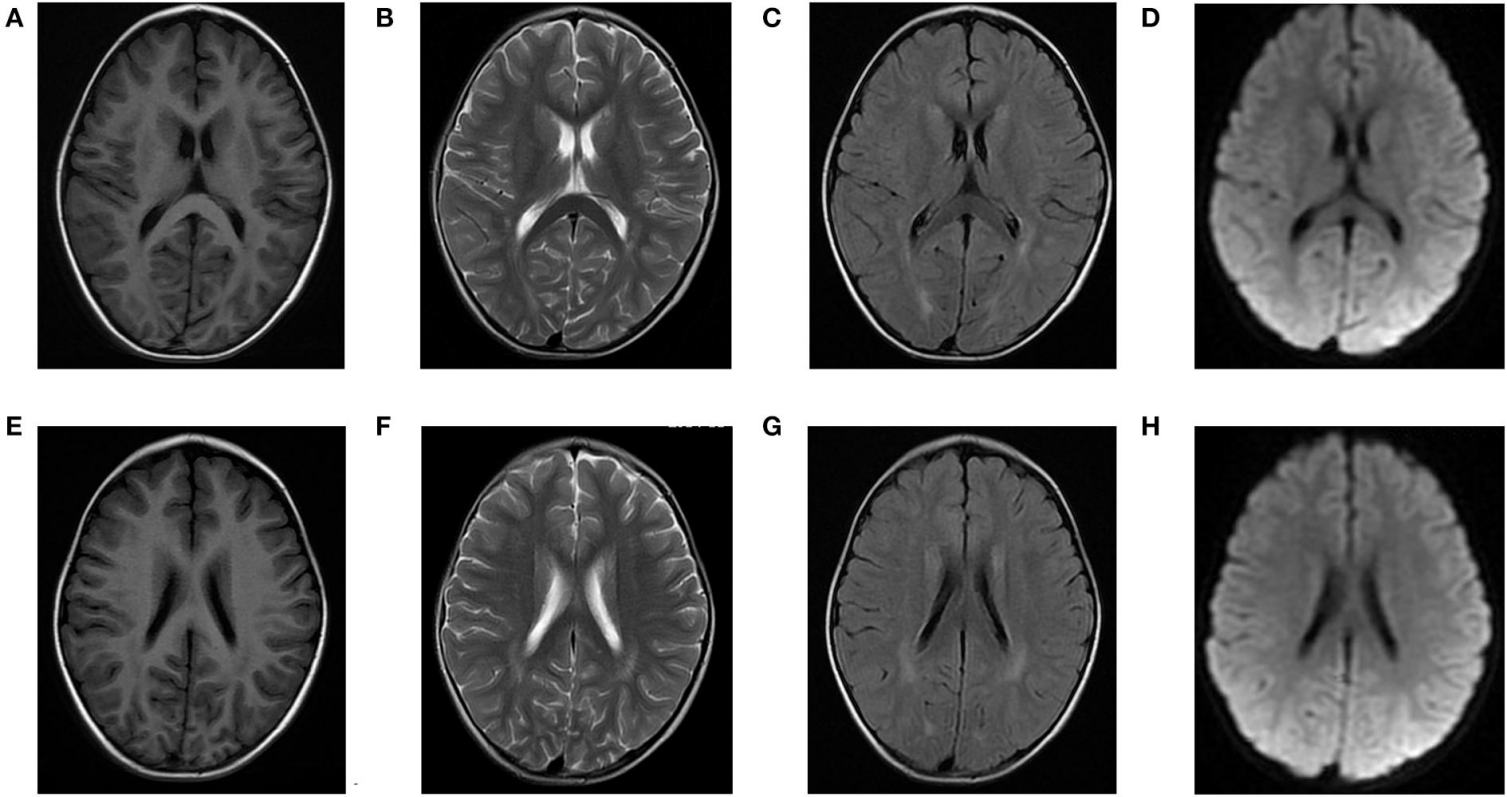
Brain magnetic resonance (MR) images of the patients. **(A–D)** and **(E–H)** show scattered high signal intensity in the bilateral frontal and parietal periventricular white matter.

**Figure 2:**
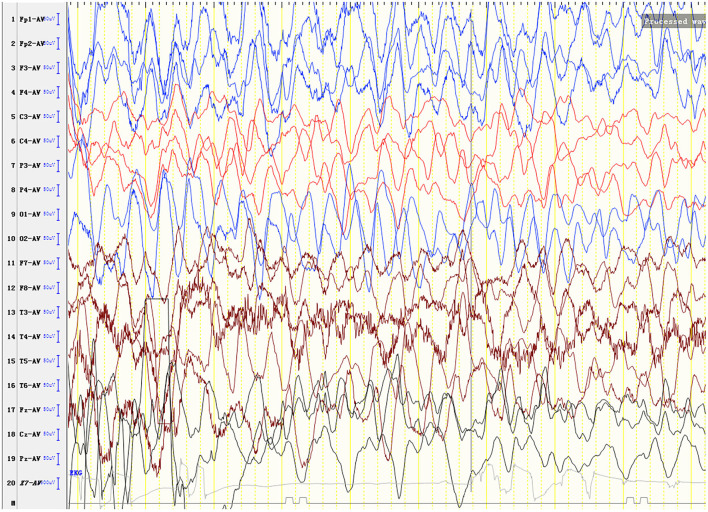
An electroencephalogram (EEG) shows diffuse slow wave activity with mainly 2–4 Hz moderate- and high-potential δ/θ activity, approximately symmetrical.

Laboratory analysis revealed metabolic acidosis with a blood pH of 7.28 (reference range, 7.35–7.45); bicarbonate, 10.3 mmol/L (26–32 mmol/L); blood ammonia, 160 μg/dl (25–94 μg/dl); blood glucose, 6.5 mmol/L; and uric acid, 1,246 μmol/L (90–420 μmol/L). The pressure, routine test, and biochemistry indicators of CSF were normal, and the test results of the bacterium, fungi, virus, mycoplasma of CSF were negative. Magnetic resonance imaging (MRI) revealed scattered high signal intensity in the bilateral frontal and parietal periventricular white matter (Figure 1). An electroencephalogram (EEG) showed diffuse slow wave activity with mainly 2–4 Hz moderate- and high-potential δ/θ activity, approximately symmetrical (Figure 2). Tandem mass spectrometry (MS/MS) revealed increased blood levels of C5 acylcarnitine, and Gas chromatography–mass spectrometry (GC/MS) revealed increased levels of urinary 3-hydroxyisovaleric acid (3HIV), isovalerylglycine, and urinary ketone bodies, confirming the diagnosis of IVA.

**Figure 3:**
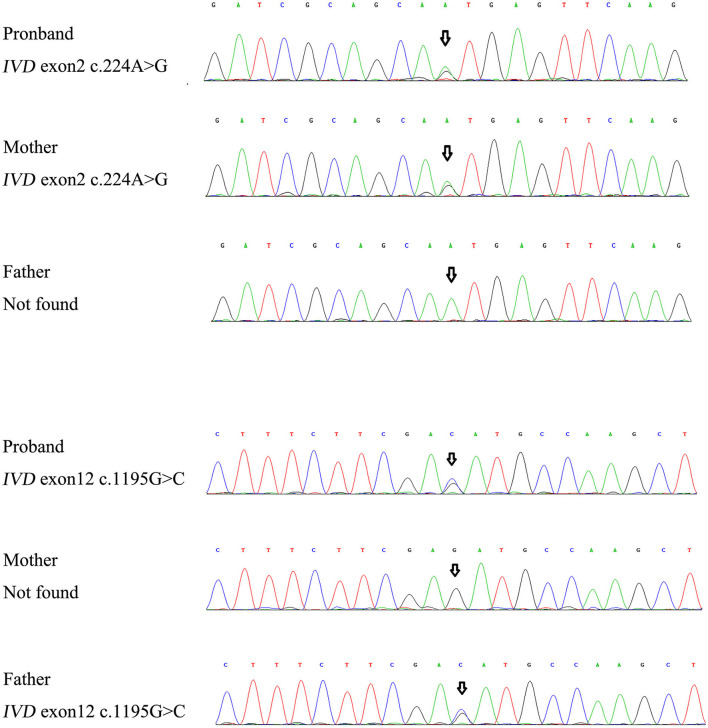
The gene variations were validated using Sanger sequencing.

**Figure 4:**
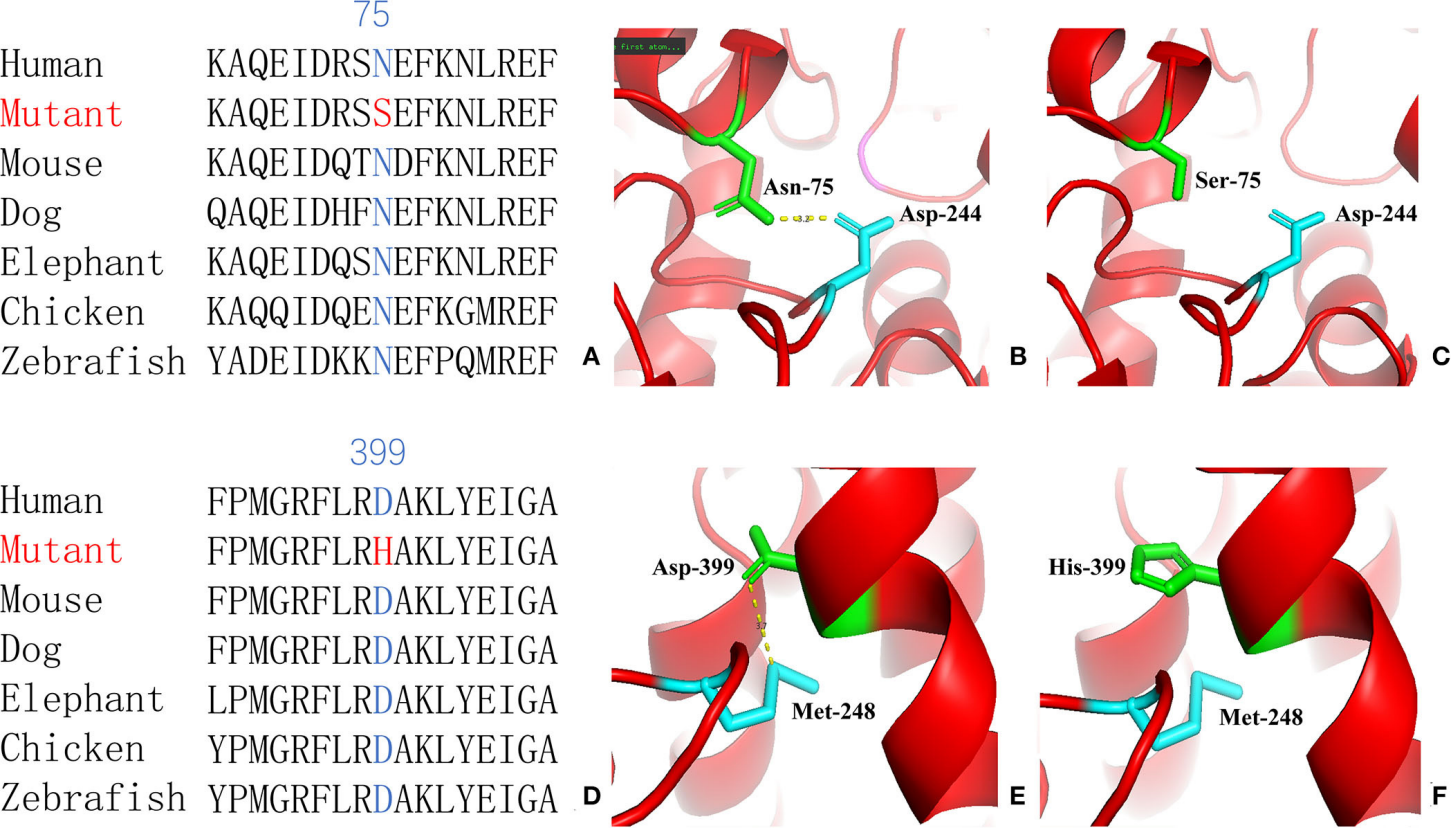
Conservation of the identified amino acids across diverse species **(A, D)** and the 3D modeling for the *IVD*-wild and identified missense variant of site 75 **(B, C)** and site 399 **(E, F)** in our patient with isovaleric acidaemia (IVA).

For genetic analysis, DNA was extracted from the peripheral blood of the patient and his parents using the QIAamp DNA Blood Mini Kit (QIAGEN). A total of 3 μg of patient DNA was cleaved into 200–300 base-pair fragments. The whole-exome enrichment was performed using an Agilent SureSelect Target Enrichment System (Agilent Technologies). Coding exons and flanking intronic regions were enriched using the Agilent SureSelect Human All-Exon V6 reagent (Agilent Technologies) according to the manufacturer's protocol. The captured libraries were loaded onto a HiSeq 2500 platform (Illumina). Base calling and assessments of sequence read quality were performed using Illumina Sequence Control Software (SCS; Illumina). Reads with average quality scores of <25 were removed, and bases with quality scores of <20 were trimmed. The mean read depth for each sample was 100× . The reads were aligned to the human reference genome (UCSC GRCh37/hg19) using the Burrows–Wheeler Aligner BWA-MEM algorithm (BWA v0.7.15). Reads with low mapping quality scores were excluded. Presumed PCR duplicates were identified and removed using the Picard MarkDuplicates. Local alignment optimisation and base quality recalibration were performed using the Genome Analysis Toolkit. Single-nucleotide variants and InDels were identified using Mutect2 and saved in a variant call format. Variants were functionally annotated and filtered using ANNOVAR (http://annovar.openbioinformatics.org/en/latest), which provides built-in public databases (OMIM, InterVar, ClinVar, HGMD, Cosmic70, dbSNP, 1000G, ESP, ExAC, and gnomAD) and the HGMD professional database. We identified two compound heterozygous variants of the *IVD* gene, the unreported variant c.224A>G (p.Asn75Ser) and the reported variant c.1195G>C (p.Asp399His). The gene variations were validated using Sanger sequencing (Figure 3). The conservation of the identified amino acids across multiple species and the 3D protein modeling of the variants are shown in Figure 4. The pathogenicity scores were determined using online tools, such as Polyphen2, SIFT, Mutation Taster, and ACMG (American College of Medical Genetics) classification (Table 1).

**Table 1:** The pathogenicity predicted using online tools.

**Nucleotide change**	**Amino acid change**	**Parental derivation**	**Pathogenicity prediction**			
			**Polyphen2 (score)**	**SIFT** **(score)**	**Mutation taster**	**ACMG**
c.224A>G	p.Asn75Ser	Maternal	Probably damaging (0.918)	Deleterious (−4.485)	Disease causing	Likely pathogenic PM2_supporting, PM3 PP2, PP3, PP4
c.1195G>C	p.Asp399His	Paternal	Probably Damaging (1.000)	Deleterious (−6.933)	Disease causing	Likely pathogenic PM1, PM2_supporting PM3, PP3, PP4

### Treatment and follow-up

The child was prescribed a low-protein diet (0.5 g/kg/day) supplemented with L-carnitine (10 mg/kg/day). During the 8-year follow-up, despite the need to take daily medication and resist gourmet deals, no metabolic disorder or encephalopathic symptoms recurred. At present, the child is 11 years of age and has normal mental and motor performance at present. GC/MS revealed increased levels of isovalerylglycine, and no other products were found.

## Literature review

We searched for reported cases in the PubMed database and the Wanfang database of China using the term “isovaleric acidaemia”. A total of 154 cases from 25 relevant references were combined with the present case (3, 4, 9–31), resulting in a sample of 155 patients, comprising 52 asymptomatic individuals and 103 symptomatic individuals (including 64 with neonatal onset, and 39 with chronic intermittent disease) with onset from ages from 1 month to 10 years (median age, 2 years).

### Clinical data summary

#### Clinical manifestations

Clinical manifestations included vomiting (50.5%, 52/103), lethargy (42.7%, 44/103), “sweaty foot” odor (37.9%, 39/103), poor feeding (29.1%, 30/103), developmental delay (27.2%, 28/103), epilepsy (9.8%, 10/103), dystonia (7.8%, 8/103), and tachypnoea (9.8%, 10/103). The prevalence of clinical manifestation of different clinical types is listed in Table 2, with no gender difference. The prevalence of vomiting in the chronic intermittent type was higher than that in the acute neonatal type [69.0 (27/39) vs. 39.0% (25/64)], and the prevalence of sweaty foot odor in the chronic intermittent type was lower than that in the acute neonatal type [23.1 (9/39) vs. 46.9% (30/64)]. There was no statistical difference in other clinical manifestations between the two types.

**Table 2:** The clinical characteristics of 155 patients with isovaleric acidaemia (IVA).

**Clinical data/The regions of the mutations**		**The symptomatic group**	**The asymptomatic group**	***P*-value**	**The acute neonatal group**	**The chronic intermittent group**	***P*-value**
Age at onset		2y (1m to 10y)	Neonatal-Adult	/	Neonatal	2y (1m to 10y)	/
Gender	Male	48	16	1.000	33	15	0.302
	Female	52	16		30	22	
The last	Survive	56	52	0.001	28	28	0.001
follow-up	Death	12	0		12	0	
Clinical Data	Vomiting	50.5% (52/103)	—	/	39.0% (25/64)	69.0% (27/39)	0.004
	Lethargy	42.7% (44/103)	—	/	46.9% (30/64)	35.9% (14/39)	0.310
	Sweaty foot odor	37.9% (39/103)	—	/	46.9% (30/64)	23.1% (9/39)	0.021
	Poor feeding	29.1% (30/103)	—	/	34.4% (22/64)	20.5% (8/39)	0.180
	psychomotor delay	27.2% (28/103)	—	/	26.6% (17/64)	28.2% (11/39)	1.000
	Epilepsy	9.8% (10/103)	—	/	14.1% (9/64)	2.6% (1/39)	0.085
	Dystonia	7.8% (8/103)	—	/	10.9% (7/64)	2.6% (1/39)	0.253
	Tachypnea	9.8% (10/103)	—	/	9.4% (6/64)	10.3% (4/39)	1.000
Auxiliary examination	Metabolic acidosis	44.7% (46/103)	—	/	43.8% (28/64)	46.2% (18/39)	0.840
	Hyperammonemia	38.9% (40/103)	—	/	10.9% (7/64)	23.1% (9/39)	0.159
	Ketosis	15.5% (16/103)	—	/	45.3% (29/64)	28.2% (11/39)	0.098
	Cytopenia	25.2% (26/103)	—	/	29.7% (19/64)	17.9% (7/39)	0.244
	Pathoglycemia	8.7% (9/103)	—	/	7.8% (5/64)	10.3% (4/39)	0.727
	Isovalerylglycine/ isovalerylcarnitine	100% (78/78)	91.7% (44/48)	0.019	100% (50/50)	100% (28/28)	/
Genetic analysis	Homozygous	54.2% (52/96)	34.7% (17/49)	0.035	53.4% (31/58)	55.3% (21/38)	1.000
	Compound heterozygous	45.8% (44/96)	65.3% (32/49)	0.035	46.6% (27/58)	44.7% (17/58)	1.000
	The 123-159 Region	23.4% (33/141)	4.7% (4/85)	0.000	25.6% (22/86)	20.0% (11/55)	0.542
	The 282-318 Region	5.0% (7/141)	23.5% (20/85)	0.000	4.7% (4/86)	5.5% (3/55)	1.000
	The 356-403 Region	31.2% (44/141)	18.8% (16/85)	0.044	29.1% (25/86)	34.5% (19/55)	0.577

On auxiliary examination, abnormal findings included metabolic acidosis (44.7%, 46/103), hyperammonaemia (38.9%, 40/103), ketosis (15.5%, 16/103), cytopenia (25.2%, 26/103), and abnormal blood glucose levels (8.7%, 9/103). Isovalerylglycine/isovalerylcarnitine/3-hydroxyisovaleric acid eluted at 97% (122/126), and the C5-carnitine level was increased in 100% (112/112). There was no statistical difference in the auxiliary examination between the acute neonatal type and the chronic intermittent type (Table 2).

#### Prognosis

At the last follow-up, 68 patients were mentioned, including 56 survived and 12 died. The 56 surviving patients had a median age of 8 years (range, 1–25 years) and a median follow-up of 8 years (range, 2–22 years). In total, 34 cases developed normally without other complications; 22 cases had developmental/mental impairment, other neurological disorders, and paroxysmal metabolic crises; and 12 died, all of whom were acute neonatal type, and seven of them died during the neonatal period. There was no death in the chronic intermittent type, and the difference was statistically significant (*p* < 0.05) (Table 2).

#### Genetic analysis

Compound heterozygous and homozygous variants were present in 52.4 (76/145) and 47.6% (69/145), respectively. A total of 226 *IVD* variants were identified, including 213 missense variants, 7 deletion variants (2 large fragment deletions), 4 insertion variants, and 2 insertion-deletion variants. There were 197 exon variants and 29 intron variants. The proportion of homozygous mutations in symptomatic type (such as acute neonatal type and chronic intermittent type) was higher than that in asymptomatic type [54.2 (52/96) vs. 34.7% (17/49)], and the proportion of compound heterozygous mutations in the asymptomatic type was higher than that in the symptomatic type [65.3 (32/49) vs. 45.8% (44/96)]. The difference was statistically significant (*p* < 0.05), but there was no statistical difference between the acute neonatal type and the chronic intermittent type (Table 2).

### Correlation between domains and phenotypes in the distribution of pathogenic variants

**Figure 5:**
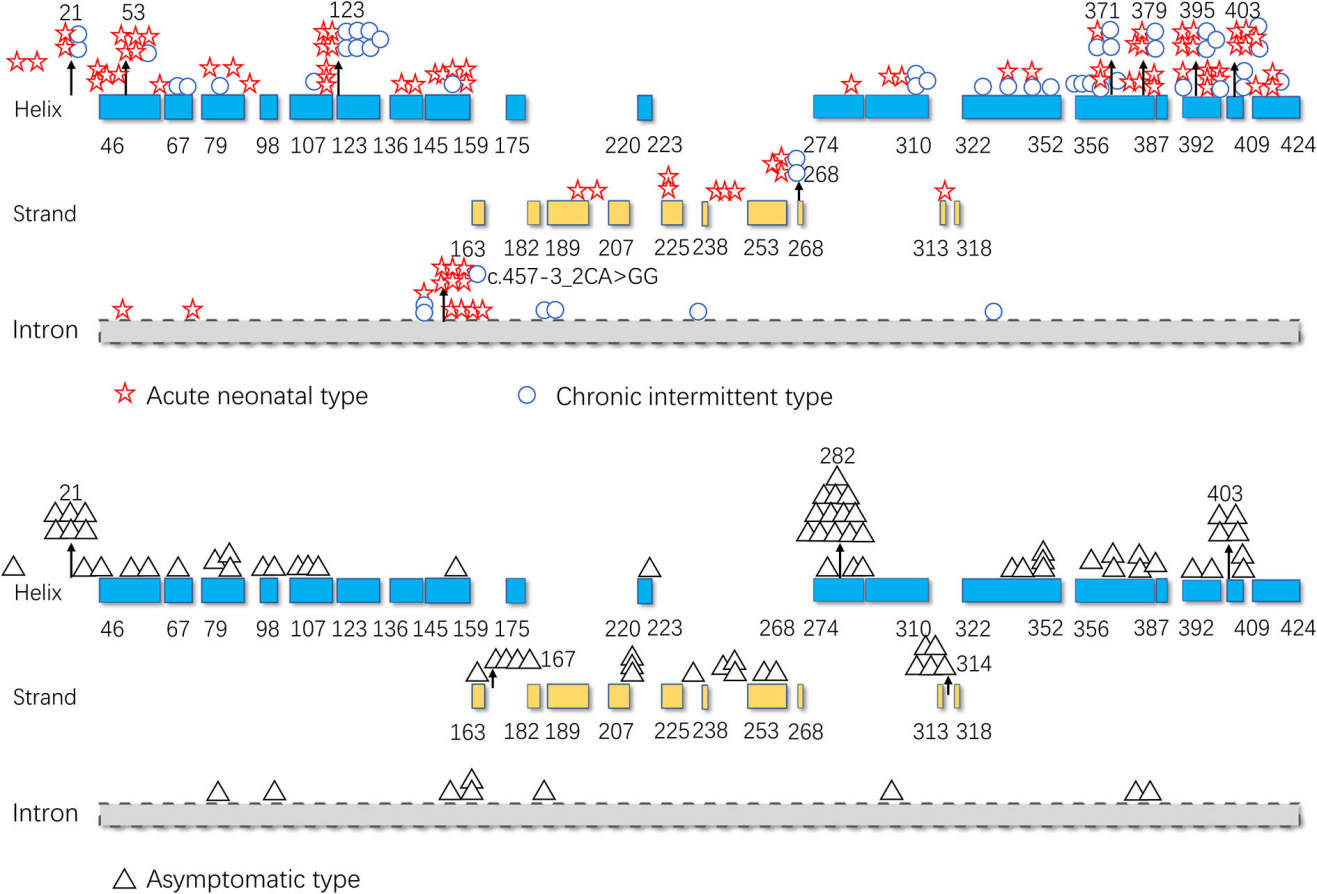
Distribution of the isovaleryl-CoA dehydrogenase *(IVD)* variants. Blue: the α-helical N-terminal domain and the second α-helical C-terminal domain of *IVD;* yellow: the β-sheet domain; and gray: intron region. Numbers represent the corresponding amino acid sites.

As illustrated in Figure 5, pathogenic variants were non-uniformly distributed along the *IVD* sequence. Therefore, to further determine the correlation between the genotype and phenotype of *IVD*, we applied cumulative distribution functions for the first time to analyze whether the pathogenic variants were clustered in any specific regions.

**Figure 6:**
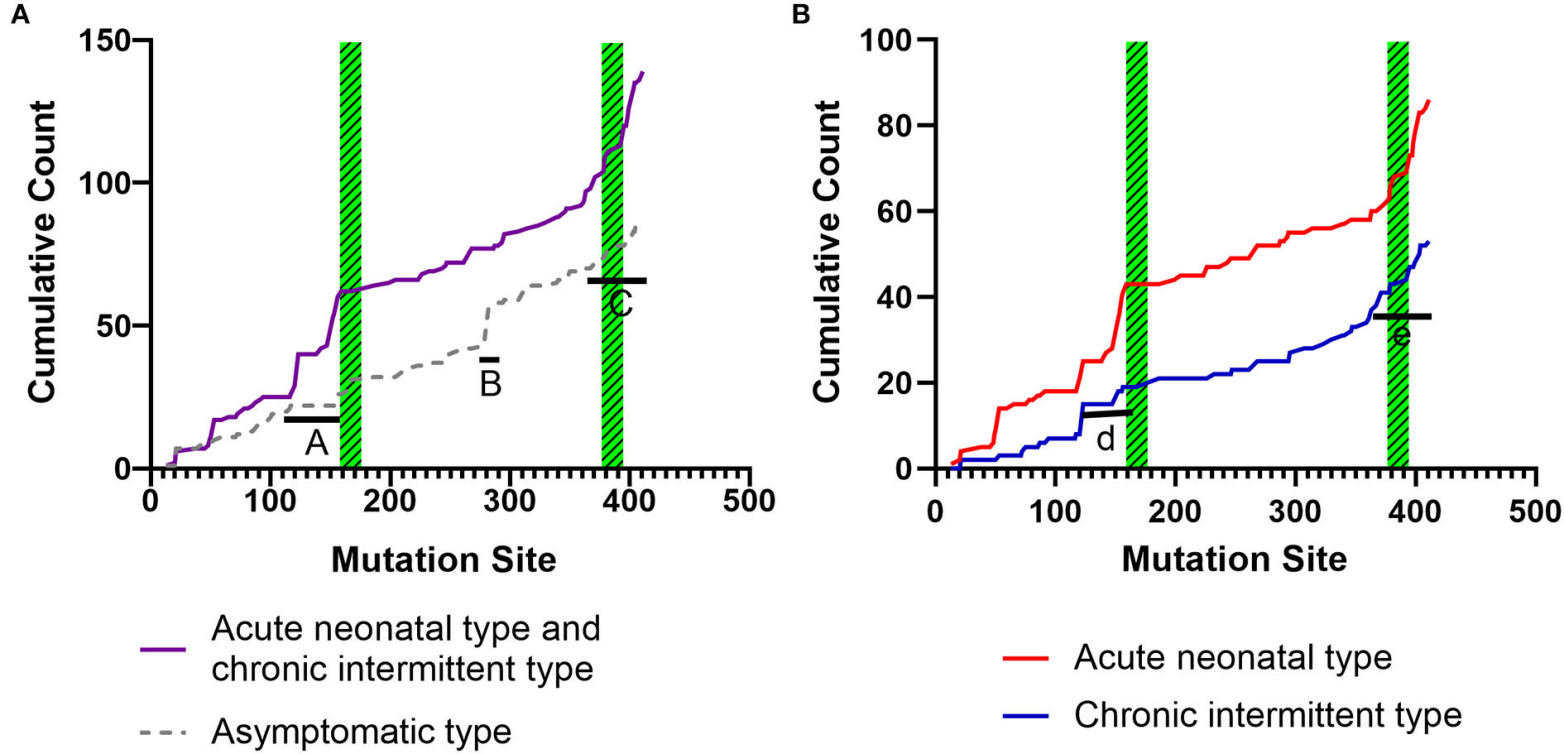
Cumulative distribution of the *IVD* variants, comparing variant distribution in symptomatic patients (including, acute neonatal and chronic intermittent type) with that of asymptomatic patients **(A)**, and the acute neonatal type vs. the chronic intermittent type **(B)**. Green striped boxes: FAD binding sites. FAD, flavin adenine dinucleotide.

**Figure 7:**
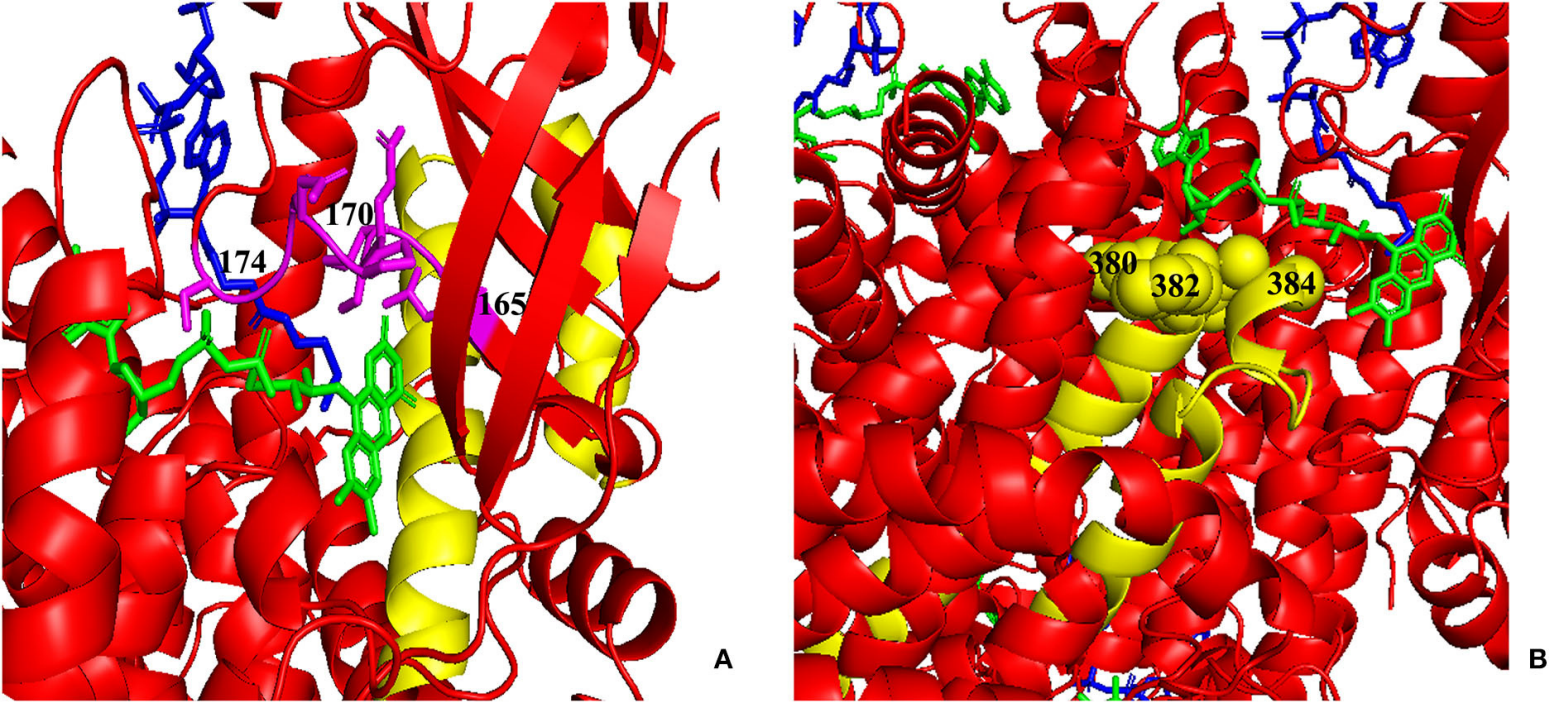
Three-dimensional (3D) modeling of areas 123–159 and 356–403. **(A)** Yellow: the 123–159 region; green: FAD; blue: CoA Persulfide; purple: FAD binding sites (numbered). **(B)** Yellow: the 356–403 region; green: FAD; blue: CoA Persulfide; yellow balls: FAD binding sites (numbered). FAD, flavin adenine dinucleotide.

**Figure 8:**
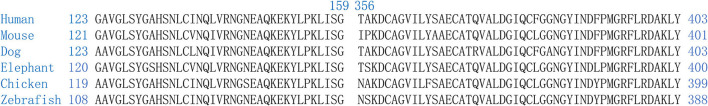
Conservation of amino acid sequences in areas 123–159 and 356–403 across diverse species.

Figures 6A,B shows the cumulative distribution of symptomatic patients (including acute neonatal and chronic intermittent types) and asymptomatic, acute neonatal, and chronic intermittent patients, respectively. In Figure 6A, three steep regions were revealed: (a) 123–159; (b) 282–318; and (c) 356–403. In each of the three steep regions, we compared the difference in the number of variants between symptomatic and asymptomatic patients, and the results showed that the proportion of variants was higher in symptomatic patients than in asymptomatic patients in regions 123–159 and 356–403 (*p* < 0.05 and *p* < 0.05). In the 282–318 region, the proportion of asymptomatic patients was greater than that of symptomatic patients (*p* < 0.05) (Table 2). In Figure 6B, two steep areas can be observed: (a) 123–159 and (b) 356–403. Within each steep region, we observed no difference in the ratio of the number of variants in patients with the acute neonatal type and in those with the chronic intermittent type (*p* > 0.05 and *p* > 0.05) (Table 2). The 3D modeling and conservation of amino acid sequences among different species in areas 123–159 and 356–403 are shown in Figures 7, 8, respectively.

### Distribution of the *IVD* variants in chinese patients

**Figure 9:**
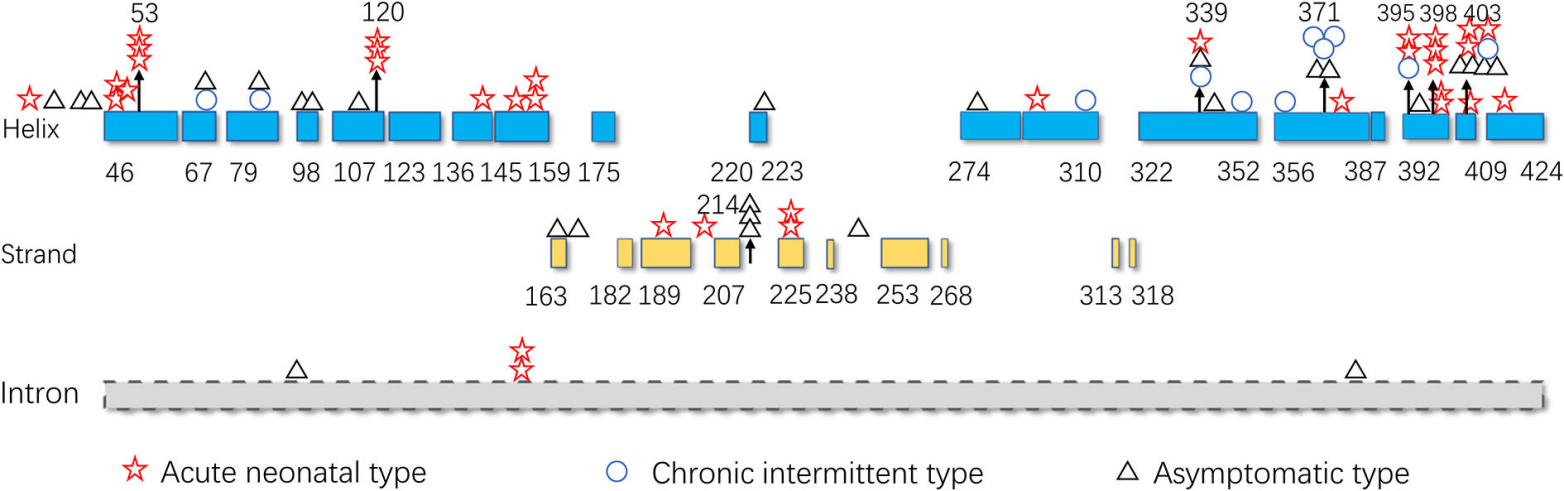
The *IVD* variant distribution in Chinese patients with IVA.

The hot region of the distribution of *IVD* variants in Chinese patients with IVA was 356–403, and the hotspots were 53, 120, 214, 339, 371, 395, 398, and 403 (Figure 9).

## Discussion

Isovaleric acidaemia is one of four typical organic acidaemias: propionic acidaemia, methylmalonic acidaemia, glutaric acidaemia, and IVA. Currently, there are no data on the overall prevalence of IVA in China. The prevalence of neonatal IVA is 1:400,000 in the neonatal screening data of the Shanghai Institute of Pediatrics (25). The overall prevalence of IVA is 1:365,000 in Taiwan (9), 1:250,000 in the United States (26), and 1–9/100,000 in other Western countries (21).

The structure of IVD was first elucidated in 1966, and the enzyme was first isolated and purified from rat-liver mitochondria in 1983 (2). It is a homologous tetramer that belongs to an important family of flavin proteins. Each monomer consists of three domains: an α-helical domain at the N-terminus, a β-sheet domain, and a second α-helical domain at the C-terminus. The α-helical N-terminal domain consists of six α-helices labeled A–F from the N-terminus toward the C-terminus. The β-sheet domain comprises seven β-sheets labeled 1–7. The C-terminal α-helical domain consists of five α-helices designated G–K. Each monomer contains an FAD molecule (also known as active vitamin B2) that catalyses the α- and β-dehydrogenation of various thioester substrates. The opening of the CoA binding pocket, where the adenosine diphosphate portion of CoA binds, is exposed to the solvent, while the bottom of this pocket, which surrounds the fatty acyl segment of the CoA thioester substrate, is mainly composed of hydrophobic amino acid residues of the polypeptide and the isoalloxazine ring of FAD. The residues that line the substrate binding pocket and the active site cavity are located on the loops connecting β-strands 1 and 2, β-strands 4 and 5, and the loops between helices J and K, H and I, and E and G (2).

IVD catalyses the oxidation of isovaleryl-CoA to 3-methylcrotonyl-CoA during the third step of leucine catabolism. IVD deficiency disrupts leucine metabolism, leading to the accumulation of upstream isovaleric acid and its metabolites 3-hydroxyisovaleric acid, isovaleryl (C5)-carnitine, and isovalerylglycine (11), resulting in the metabolic acidosis and functional impairment of the brain, liver, kidneys, bone marrow, and other organs.

To date, the correlation between phenotype and genotype in patients with IVA has been unclear, and siblings carrying the same variant have shown very different phenotypes (17). Therefore, in this study, the data from 155 patients were summarized, and the correlation between phenotype and genotype was further analyzed. The results showed that the two hot regions of the distribution of pathogenic variants in symptomatic patients (including, acute neonatal type and chronic intermittent type) were located in the 123–159 and 356–403 regions, while the distribution in asymptomatic patients was mainly located in the 282–318 region. The c.932C>T (p.Ala282Val) and c.941C>T (p.Ala314Val) variants were common in asymptomatic and neonatal screening patients who remained asymptomatic during the follow-up period. This indicates that the variants in the first two regions are highly pathogenic, whereas the concentrated distribution of variants with an asymptomatic phenotype indicates that the amino acid variations are pathogenically milder.

As shown in Figures 5, 7, the 123–159 and 356–403 regions were mainly in the α-helix of the N-terminal domain near the center of the monomer and in the α-helix at the end of the C-terminal domain, respectively, which are relatively conserved across diverse species (Figure 8). The C-terminal amino acids of IVD are necessary for tetramer stability and subunit interaction. According to the Uniprot database, regions 165–174 and 380–384 are FAD binding sites. FAD has an extended conformation and is located between the middle and C-terminal domains of one monomer and the C-terminal domain of the adjacent monomer (2). As shown in Figure 6, the aggregated regions of the pathogenic variant distribution were essentially near the FAD binding sites, with partially overlapping regions. Therefore, variants in these two regions may affect the binding between amino acid residues and FAD molecules, destroy the protein structure, affect the stability of the tetramer, and further affect protein function. Asymptomatic hot regions are located at 282–318, which is far from the FAD binding sites; therefore, they may have little influence on protein function.

Our patient was identified as having the compound heterozygous variants c.224A>G (p.Asn75Ser) and c.1195G>C (p.Asp399His). Asn75 is located in the α-helical N-terminal domain, whereas Asp399 is located in the α-helical C-terminal domain. All these residues are embedded in the protein and interact with other residues to stabilize the protein structure. When Asn75 is replaced with serine, the hydrogen bond between Asn75 and Asp244 is predicted to be disrupted (Figure 4), thus affecting protein structure and stability. When Asp399 is replaced with histidine, an imidazole ring is added to the side chain, resulting in destroying the hydrogen bond between Asp399 and Met248 (Figure 4), which changes three-dimensional structure and may affect the binding of FAD.

The hotspots for pathogenic variants depend on ethnicity and region. According to previous reports, c.1199A>G (p.Tyr371Cys) and c.1208A>G (p.Tyr403Cys) are common among the Han Chinese population (17). The variant c.466-3_466-2CA>GG (originally reported as c.457-3_2CA>GC) is common in both Thai and Korean populations (4, 22); c.932C>T (p.Ala282Val) has been reported as a recurrent variant in White populations (26); c.367G>A (p.Gly123Arg) has been described as a common variant in Whites settled in South Africa (15); c.158G>C (p.Arg53Pro) was the most common variant in the Mexican population (27); and p.Arg395Gln may be a common variant in the UAE population (23). Based on the literature review, the hotspots in the mainland China and Taiwan are 53, 120, 214, 339, 371, 395, 398, and 403 (Figure 9).

For IVA treatment, daily protein intake should be limited, and the dietary supplementation with L-carnitine and glycine should be prescribed to convert isovaleric acid to non-toxic isovalerylcarnitine and isovalerylglycine, respectively (11).

The clinical manifestations and prognosis of IVA vary greatly, and some patients die in the neonatal period. However, many asymptomatic patients are found through family or neonatal screening and remain asymptomatic during the follow-up. In the last follow-up, 12/68 had died and 56/68 had survived, 34 of whom developed normally without other complications. In total, twenty-two cases had developmental/mental impairment, other neurological disorders, and paroxysmal metabolic disorders. Early diagnosis and treatment have been reported that cannot prevent the onset of metabolic crisis but can prevent mental impairment (21). Therefore, the correlation between genotype and phenotype can help us understand the disease and guide genetic counseling, and early screening and diagnosis as early as possible for IVA can guide treatment and improve prognosis.

## Data availability statement

The datasets presented in this article are not readily available because of ethical and privacy restrictions. Requests to access the datasets should be directed to the corresponding author/s.

## Ethics statement

The studies involving human participants were reviewed and approved by Medical Ethics Committee of Tianjin Children's Hospital/Tianjin University Children's Hospital. Written informed consent to participate in this study was provided by the participants' legal guardian/next of kin. Written informed consent was obtained from the minor(s)' legal guardian/next of kin for the publication of any potentially identifiable images or data included in this article.

## Author contributions

XingL and WF retrieved and summarized the related literature. XinqL drew all the figures and tables. ZZ provided statistical analysis. PZ, XiaoL, ML, and QL followed up the patients. XY and DL revised the review article. All authors contributed to the article and approved the submitted version.

## Conflict of interest

The authors declare that the research was conducted in the absence of any commercial or financial relationships that could be construed as a potential conflict of interest.

## Publisher's note

All claims expressed in this article are solely those of the authors and do not necessarily represent those of their affiliated organizations, or those of the publisher, the editors and the reviewers. Any product that may be evaluated in this article, or claim that may be made by its manufacturer, is not guaranteed or endorsed by the publisher.
